# Syngas Fermentation to Acetate and Ethanol with Adaptative Electroactive Carboxydotrophs in Single Chambered Microbial Electrochemical System

**DOI:** 10.3390/mi13070980

**Published:** 2022-06-21

**Authors:** Athmakuri Tharak, S. Venkata Mohan

**Affiliations:** 1 Bioengineering and Environmental Science Lab, Department of Energy and Environmental Engineering, CSIR-Indian Institute of Chemical Technology (CSIR-IICT), Hyderabad 500007, India; tharakathmakuri527@gmail.com; 2 Academy of Scientific & Innovative Research (AcSIR), Ghaziabad 201002, India

**Keywords:** microbial fuel cell, electro/gas fermentation, DNA library, 16S V3–V4 amplicon, electro kinetics

## Abstract

Microbial electrosynthesis system (MES; single-chambered) was fabricated and evaluated with carbon cloth/graphite as a working/counter electrode employing an enriched microbiome. Continuous syngas sparging (at working electrode; WE) enabled the growth of endo electrogenic bacteria by availing the inorganic carbon source. Applied potential (−0.5 V) on the working electrode facilitated the reduction in syngas, leading to the synthesis of fatty acids and alcohols. The higher acetic acid titer of 3.8 g/L and ethanol concentration of 0.2 g/L was observed at an active microbial metabolic state, evidencing the shift in metabolism from acetogenic to solventogenesis. Voltammograms evidenced distinct redox species with low charge transfer resistance (R_ct_; Nyquist impedance). Reductive catalytic current (−0.02 mA) enabled the charge transfer efficiency of the cathodes favoring syngas conversion to products. The surface morphology of carbon cloth and system-designed conditions favored the growth of electrochemically active consortia. Metagenomic analysis revealed the enrichment of phylum/class with Actinobacteria, Firmicutes/Clostridia and Bacilli, which accounts for the syngas fermentation through suitable gene loci.

## 1. Introduction

Climate change issues, biosphere alteration and biodiversity loss are some of the critical challenges [1,2,3,4]. Some industrial activities release synthesis gas composed of carbon monoxide (CO), carbon dioxide (CO_2_) and hydrogen (H_2_) in different fractions [5,6,7,8]. Varies conventional strategies such as capture and storage are employed to mitigate the greenhouse gas (GHG) emissions [3,8,9,10]. Microbial mediated process of carbon sequestration, which is considered a potential strategy, can also play a crucial role in this regard [11,12]. Syngas fermentation through the Wood–Ljungdahl pathway (WLP) owned microbes contributes to the synthesis of short-chain carboxylic acids [9,13] and can sustain the toxic effects of CO [14]. However, desired product synthesis from the crude syngas fermentation is still challenging [15,16,17]. Slow fermentation rates, difficulty in threshold barrier breakdown, inconsistency in metabolic action of biocatalyst, restricted efficiency of WLP at higher CO concentrations and slow gas–liquid mass transfer due to the low solubility of CO are some of the limitations to the syngas fermentation [18,19]. Microbial electrochemical systems (MESs) mediated syngas conversion facilitates electrochemically assisted fermentation for the synthesis of metabolic products from gaseous feedstock [19,20]. Integration of inert conductive material (electrode) and electric poised conditions can augment additional gas conversion rates in the gas electro fermentation [14,20].

Several acetogenic and solventogenic carboxydotrophs utilize the C1 fraction of the syngas and produce metabolites (acetate and ethanol) [5,18]. As fermentation is influenced by the activity of microbes, selection and adaptation of the suitable biocatalyst is a critical factor. Gas phased electrofermentation with adequate biocatalyst activity provides feasibility to the syngas fermentation with the synthesis of the value-added compounds. Adaptation of the biocatalyst to the electrode-circuit environment is one major factor that influences syngas electrofermentation. The current study was designed to evaluate the single-chambered system for syngas transformations to metabolites, specifically using syngas-enriched cultures as biocatalysts. Continuous gaseous feeding with semi-continuous mode operation was employed. Fermentation efficiency was assessed with biological and bioelectrochemical parameters as well as product formation. The fate of the inorganic carbon fraction in the syngas was traced and depicted for the conversion abilities of the system.

## 2. Materials and Methods

### 2.1. Design and Fabrication of MES

**Figure 1:**
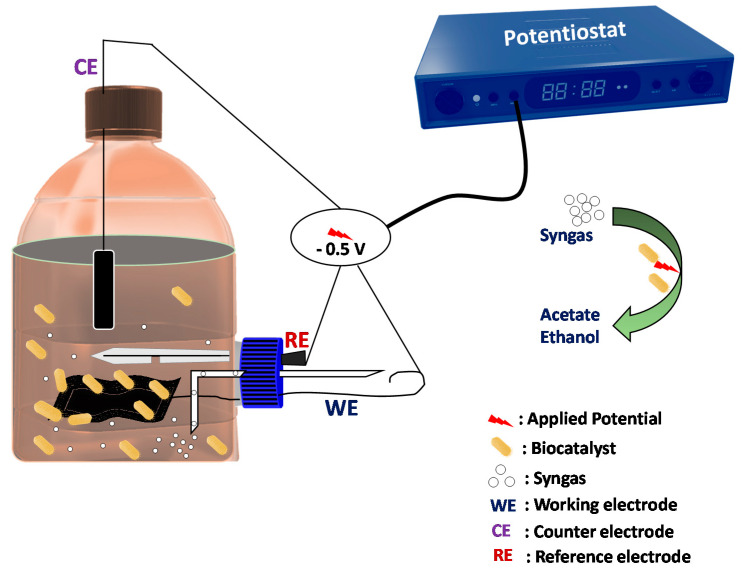
Diagrammatic representation of the single-chambered MES.

Single-chambered microbial electrosynthesis system (electrofermentation) was designed and fabricated with electrodes (inert) as the primary components (Figure 1). A screw cap glass bottle (Borosilicate^®^, Mumbai, India) was used with a working/total volume of 85/100 mL. Three electrodes in the system were arranged by using a butyl rubber stopper. The graphite plate (surface area 28 cm^2^; counter electrode, CE) was placed vertically through the rubber stopper. Ag/AgCl (3.5 M KCl) and carbon cloth (CC; 36 cm^2^) were employed as a reference, and working electrodes were situated perpendicularly to CE through the lateral port of the system. Both the working and reference electrodes were situated at ~1.5 cm distance.

### 2.2. Microbiome, Electrolyte and Feed Stock

Selectively enriched carboxydotrophic microbiome from the previously operated lab-scale C1 gas fermenter was used as seed culture in the MES [3]. Culture (10%; *v*/*v*) was inoculated prior to the experimental operation. Phosphate buffer (0.1 M; pH 7.0 ± 0.02) was used as the electrolyte. Gaseous feedstock (Syngas) of composition CO (35%), CO_2_ (30%), H_2_ (20%) and N_2_ (13.3%) were sparged into the system as an inorganic carbon source during experimental operation.

### 2.3. System Operation

**Figure 2:**
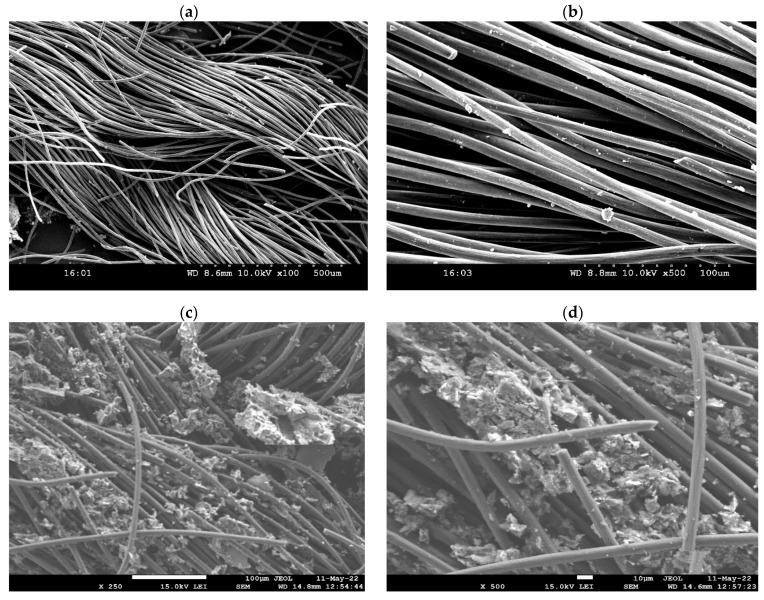
SEM images representing the working electrode morphology (Carbon clothe) before operation (abiotic): (**a**) ×100 and (**b**) ×500; after operation (biofilm): (**c**) ×250 and (**d**) ×500.

Selected consortia were adapted to the polarized environment by poising the potential (−0.5 V) on electrodes. After four feed replacements, attachment of cell was observed on carbon cloth electrodes by forming biofilm (Figure 2), which was also observed with the naked eye. The formation of biofilm on the surface of the electrodes and the growth of the suspension cells increases the turbidity of the electrolyte, which can be considered as the adaptation of the inoculated biocatalyst in the polarised environment. After the adaptation, carbon cloth was used as a working electrode in the single-chambered MES. Further MES was operated by poising the potential (−0.5 V) on the working electrode against the reference electrode. In order to ensure and assist the growth of biocatalyst, semi-continuous batch mode operation with continuous syngas feeding at a rate of 0.3 standard cubic centimeter per minute (sccm) was employed. Syngas feeding was performed near the interspace of the working electrode for effective availability of the inorganic carbon to the culture. MES was operated for a total of 6 cycles with 96 h of retention time.

### 2.4. Performance Analysis

Fermentation efficiency of MES was monitored in terms of metabolic process parameters such as pH and volatile fatty acids (VFA) by sampling electrolytes once in 24 h. The pH of the electrolyte was evaluated using the pH meter (Hanna Instruments, Woonsocket, RI, USA), and the fermentation product (VFA) profile was assessed with high-performance liquid chromatography (HPLC) system (Shimadzu LC20A, Santa Clara, CA, USA) with a refractive index detector (RID20A; Shimadzu, Santa Clara, CA, USA) employing RezexTM RHM-Monosaccharide H+ column (Phenomenex, Kondapur, India). Samples were diluted and subjected to filtration (0.2 micron) prior to HPLC analysis. Elution at a rate of 0.5 mL/min in isocratic mode was employed by using ultrapure water as the eluent (mobile phase) with 20 µL of injected sample volume [3,10].

The growth of the culture in the electrolyte was measured by using a UV-visible spectrophotometer (Jasco V-750, Europe, Cremella, Italy) at optical density (OD_600nm_). The surface morphology of the carbon cloth (WE) was studied before and after the experiments using the FE190 SEM (JOEL, JSM-7610F, Tokyo, Japan) with a voltage range of 1 to 15 kV. The gold coating was performed on the sample stub before the SEM analysis for better visibility.

### 2.5. Electrochemical Characteristics

MES was connected to the potentiostat (EC-lab, Biologic VMP3, Seyssinet-Pariset, France) with a continuous poising of −0.5 V on the WE (vs. Ag/AgCl). The electrochemical response of the biocatalyst was evaluated through electrochemical techniques such as cyclic voltammetry (CV), chrono amperometry (CA), differential pulse voltammetry (DPV) and electrochemical impedance spectroscopy (EIS). CVc and LSV were performed with a scan rate of 10 mV/s within the potential stretch of 0.8 to −1.0 V. Differential pulse voltammetry was drawn at 1st and 4th days of operation with starting potential of −1.0 V to limit potential 0.8 V followed by reverse scan to −1.0 V. A pulse height of 2.5 mV and a width of 100 mV was maintained with step height and step time of 5 mV and 500 ms, respectively. EIS measurements were conducted at the end of the experiment in the frequency range of 100 kHz to 100 mHz with the voltage amplitude of 10 mV in the open-circuit voltage (OCV) state of the system.

### 2.6. Substrate Fixation and Conversion

Fermentation efficiency of the biocatalyst was interpreted by the inorganic carbon fixation rate (ICFR; mg·L^−1^·h^−1^) and conversion efficiency of inorganic carbon (CEIC; %) to the respective fermentation products [21] using the Equations (1) and (2).

(1) $$\mathrm{ICFR}=\frac{M1-M0}{t1-t0}\times 10^{3}$$

(2) $$\mathrm{CEIC}=\frac{\left(M1-M0\right)\times V}{\begin{array}{c} Mc\\ \bar{\underline{\alpha c\times \mathrm{qv}\times \left(t1-t0\right)\times 60\times 10^{-3}}}\\ Vm \end{array}}\times 100\%$$

where M1 is the mass volume of fermentation products (TOC; g·L^−1^) at t1 (h) and M0 is the mass volume of fermentation products (g·L^−1^) at t0 (h). V represents the volume of fermentation broth (0.085 L), MC is the relative molecular mass of carbon (12.01), αc is the proportion of carbon-containing gas (65% in syngas), qv is the gas flow rate (0.3 sccm), 60 is the gas flow rate change from minutes to hours, and Vm is the molar volume of gas at standard temperature and pressure (22.4 L·mol^−1^).

### 2.7. Metagenomic Analysis

Microbial community analysis was performed by extracting the genomic DNA of the system according to the manufacturer’s protocols (DNeasy PowerSoil Pro Kit, QIAGEN, Germantown, MD, USA). Extracted DNA quality and concentration were checked by using the nano drop spectrophotometer (Thermo Scientific™, Waltham, MA, USA). Libraries of the extracted genomic DNA were performed using the Qubit DNA HS Assay Kit (Invitrogen, Carlsbad, CA, USA), and the size of the prepared library by DNA was estimated through 1000 Screen Tapes (Tapestation, Agilent, Santa Clara, CA, USA). V3–V4 regions of 16S rRNA sequence were synthesized by using the V3 forward and V4 reverse primers (P7 adapter read1 AGATCGGAAGAGCACACGTCTGAACTCCAGTCA; P5 adapter read2 AGATCGGAAGAGCGTCGTGTAGGGAAAGAGTGT). The 16S sequencing amplicon was performed by illumine Miseq 300PE plot form (Life cell International Pvt Ltd., Chennai, India). Raw sequences obtained were subjected to the FASTQC and Multi QC software (Version 0.11.9) for quality checks. High-quality reads were obtained by trimming the sequences by Trimgalore v0.6.7 with standard parameters. Filtered contigs with more than 300 bp were clustered into the operational taxonomical units (OTUs) based on the GREENGENE v.13.8-99. The abundance of OTUs was estimated to check the diversity of the microbial population.

## 3. Results

### 3.1. Productivity and Composition of Fermentation Products

**Figure 3:**
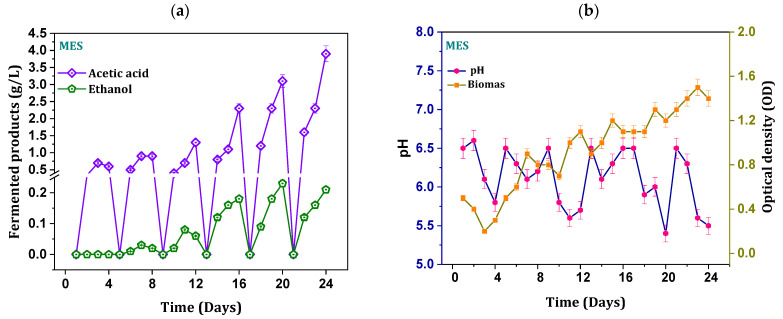
(**a**) Profile of the fermentation metabolites acetate and ethanol; (**b**) pH of the electrolyte and biomass growth with function days operated.

Metabolic fermentation product monitoring at regular intervals of time revealed the activity of the biocatalyst. Acetate production was observed after 30 h of operation during the initial batch, which persisted (0.3 ± 0.06 g/L) and increased until the end of the cycle period (Figure 3a). The observed initial lag phase in the acetate production might be due to the time required for the adaptability of the biocatalyst to the redox environment in MES. Enhancement in the biosynthesis of acetate was seen from the third day (first cycle) onwards and reached maximum titer (0.7 ± 0.02 g/L; 96 h). Microbial synthesis of the acetic acid correlates with the growth of the mixed cultures as it can utilize the acetyl-CoA reduction pathway for their reductive metabolism. Electrolyte replacement at a specific time interval favored the growth of the electroactive bacteria and facilitated an increase in the metabolic rates that resembled the biosynthesis of the syngas fermentation products. Acetic acid of 0.9 ± 0.02 g/L was produced in the second cycle, which reached a maximum of 3.82 ± 0.4 g/L during the sixth cycle with a linear increment in the number of cycles operated (3rd 1.3; 4th 2.3; and 5th 3.1 g/L). Cumulative (volumetric) productivity of acetate (1 ± 0.02 g·L^−1^·day^−1^) suggested the stabilized fermentation of the biocatalyst, which also influences the syngas conversions (Figure 3a). Though there were linear and steady increment metabolite concentrations, retention time was maintained at 96 h to overcome the potential of MES due to syngas causing an imbalance of the electron flux and sink.

An increase in the acidic charged product concentration in the electrolyte tends to imbalance the ionic transport of bacterial cell membrane, leading to cell lysis [21]. In the process of self-defense metabolism, electroactive bacteria transform the acidic charged fermentation products (acetate) into the neutrally charged fermentation products (aldehydes and alcohols) [19,21]. Similar to acetic acid, ethanol production followed steady increments after the second cycle and attained a peak during the sixth cycle (0.23 ± 0.04 g/L). As a result of the shift in the metabolism, in addition to acetic acid, ethanol production was also observed in the second cycle of operation. A relatively slow phase of ethanol synthesis was observed due to the time lap to reach the threshold accumulation of primary products (acetate). Cumulative (volumetric) ethanol production of 0.08 g·L^−1^·day^−1^ was depicted during operation. A stable maximum product titer of acetate and ethanol might indicate the adaptation of the electroactive consortia in MES. Stoichiometry of Wood–Ljungdahl pathway for syngas to acetate and ethanol is depicted in Equations (3) and (4) [21,22].

(3) $$4\mathrm{CO}+2H_{2}O+2{\mathrm{CO}}_{2}+4H_{2}\to 2{\mathrm{CH}}_{3}\mathrm{COOH}+2{\mathrm{CO}}_{2}+2H_{2}O$$

(4) $$6\mathrm{CO}+3H_{2}O+2{\mathrm{CO}}_{2}+6H_{2}\to 2{\mathrm{CH}}_{3}{\mathrm{CH}}_{2}\mathrm{OH}+4{\mathrm{CO}}_{2}+3H_{2}O$$

### 3.2. Biomass and pH

Initially maintained neutral pH of the electrolyte turned to slightly acidic pH upon experimental operation. The shift in the pH of the medium might be due to the accumulation of synthesized acetate and the acidic nature of sparged gas (CO_2_). Overall maximum pH drop (5.0 ± 0.2) was observed from the fifth cycle onwards (Figure 3b). However, irregularity in the pH profiles was noticed with sharp increased and decreased trends, which might be regulated by the water gas shift of the syngas and conversion of the acids into the alcohols in solventogenic phase. Biomass of culture at the point of inoculation was maintained at an OD of 0.5 (active log phase). A reduction in biomass growth was depicted in the first cycle of operation (Figure 3b). The reduction trend of biomass growth in initial cycles might be due to the adaptation to syngas-induced inhibitory effect and acidic fermentation product accumulation. Similar kinds of observations were reported in other studies [12,21]. Unlike acetogenic activity, solventogenic metabolism by the carboxydotrophs favors the biomass growth by converting the acid-charged molecules into neutral-charged compounds [21]. Growth of the biomass increased after ethanol production, indicating the start of solventogenic metabolism that facilitated conversion to aldehydes and finally to alcohols. The growth of the biocatalyst in the solventogenic phase was evidenced by the change in the morphology (appearance of microbial adherence) of the working electrode before and after operations (Figure 2). The attachment of biofilm on carbon cloth also depicted the system adaptation and performance.

### 3.3. Fixation and Conversion Efficiencies

**Figure 4:**
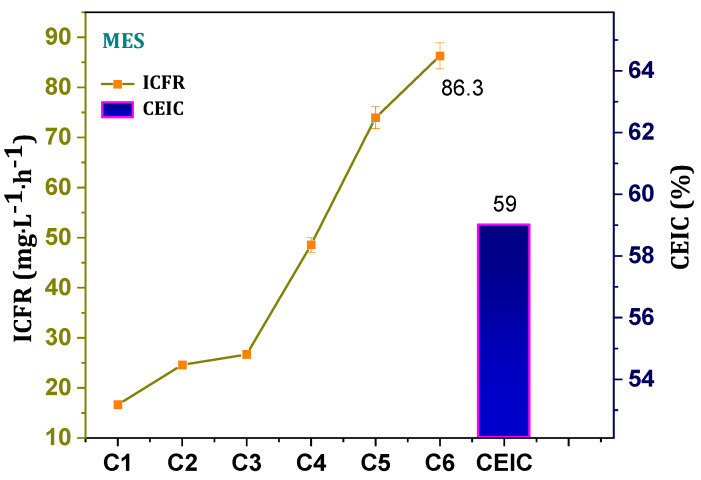
Inorganic carbon fixation rates (ICFR) with respect to the number of cycles operated and overall cumulative conversion efficiency of inorganic carbon (CEIC).

Continuous syngas feeding to the MES enhances the growth and metabolism of the gas-feeding carboxydotrophs. As the biomass growth and concentration of the acetogenic/solventogenic metabolic products increased with the number of cycles operated, the fixation rate of the inorganic carbon was also enhanced. ICFR with the given syngas (35% CO and 30% CO_2_) becomes fixed by the electrochemically active carboxydotrophs through the acetyl-CoA reduction pathway and is ultimately reduced to acetate and alcohols. ICFR of 17 mg·L^−1^·h^−1^ was observed in the first cycle, and it was followed by progressive increments with addon fixated inorganic carbon (Figure 4). A 25 and 27 mg·L^−1^·h^−1^ of ICFR was observed in the second and third cycles, respectively, revealing the enrichment and adaptation to the reduced poised potential microenvironments. Higher ICFR was observed in the sixth cycle (86 mg·L^−1^·h^−1^) with significant increment in the preceding cycles (49/74 mg·L^−1^·h^−1^; 4th/5th cycle). Continuous water and gas interaction in the electrolytes leads to a shift between gas and water, enabling the intake of gaseous feedstock into the cell prior to becoming fixed to acetyl-CoA in WLP. CEIC in the reduced electro-environment was 59% (excluding inorganic carbon fixated for biomass growth) towards the fermentation products (Figure 4). The continuous flow rate of the syngas facilitated efficient substrate availability. Higher dissipation of inorganic carbons (CO/CO_2_) in the electrolyte assists the conversion efficiencies of the electroactive biocatalyst.

### 3.4. Electrotrophy of Biocathode

**Figure 5:**
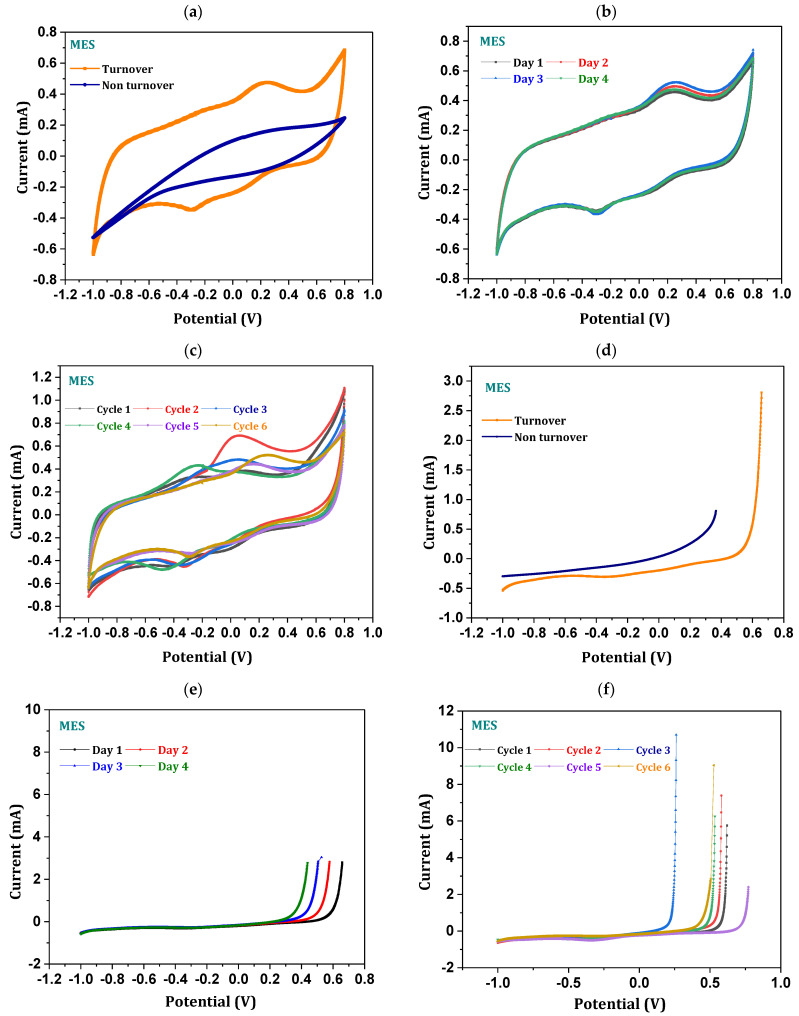
(**a**) Cyclic voltammograms (Ewe vs. Ag/AgCl (3.5 M KCl)) of the working electrode in CC-MES turnover and non-turnover comparison; (**b**) number of days operated single cycle; (**c**) comparison of each cycle cyclic voltammograms; (**d**) linear sweep voltammograms (Ewe vs. Ag/AgCl (3.5 M KCl)) of working electrode in MES turnover and non-turnover comparison; (**e**) number of days operated in single cycle; (**f**) comparison of each cycle sweep voltammograms.

Voltammograms recorded with respect to the working electrode show the faradaic electroactive species involved in the gaseous feedstock reduction into fermentation products. Pseudo electrochemical (non-faradaic) response errors were minimized and suppressed by recording the electrochemical readings of the non-turnover (with uninoculated electrolyte) condition. Non-turnover redox current (oxidative catalytic current (OCC)/reductive catalytic current (RCC)) of 0.2 mA/−0.56 mA was recorded. Non-faradaic double-layer current responses were observed with plain voltammograms with no indication of electroactive redox species presence. Non-faradaic double-layer current in the non-turnover might be due to the interaction of the working electrode and electrolyte (Figure 5a). After inoculation, redox currents (0.67/−0.71 mA) increased the voltammograms representing the electroactive redox (especially reduction) capabilities of the biocatalyst. An increase in voltammograms covering the area of turnover when compared with non-turnover showed the enhanced capacitance of the biocathode, which can be attributed to the biofilm growth on the electrode (Figure 5a) [23,24,25]. Specific peaks were observed on voltammogram at the −0.6 V and −0.49 V (vs. Ag/AgCl), representing the H_2_ evolution reaction and CO, CO_2_/acetate redox potentials, respectively. Peak intensities at −0.49 V (0.43 mA; vs. Ag/AgCl) inferred the conversion of syngas to acetate. Along with the reduction species, oxidative peaks were observed at 0.2 V, which might represent the involvement of membrane cytochromes (cytochrome c-_1_ and c-a) in an oxidized state [24,25]. Distinct oxidation peaks in the voltammograms reveal the electroactivity of the biocatalyst slimy layer on biocathode in MES [25]. No specific changes were observed in the voltammograms (for 4 days), revealing the electrochemical activity of the biocatalyst remains stable (Figure 5b). A marginal shift in the position of the redox peaks was observed with progressive cycle operations, which might be due to altering the activities of the respective redox species (Figure 5c). The structural morphology of the working electrode (carbon cloth) was observed to provide an adequate surface area for microbial biofilm formation in a shorter period of time (Figure 2).

In order to study the reduction abilities of the biocathode, linear sweep voltammetry (LSV) was recorded along with the CV. LSV (turnover) depicts higher reduction currents than the non-turnover average reduction value (−0.18 mA) (Figure 5d). A maximum of −0.6 mA (average of −0.41 mA) reduction current was noticed in the sixth cycle operation. Enhanced reduction currents in LSV suggest the syngas reduction capabilities of biocathode. Decreased oxidized stretch was observed on the LSV profile, which might be due to relatively lower counter electrode activity after the 96 h of operation (Figure 5e,f). Rapid reduction current with decreased oxidation window indicates reduction ability of the biocatalyst, which can consume the negatively charged moieties for the conversion of inorganic fraction of syngas. The formation of the fermentation product profile was correlated well with the electroactive reduction abilities of the biocatalyst observed in CV and LSV.

### 3.5. Kinetics of Charge Transfer-Biocathode Response

**Figure 6:**
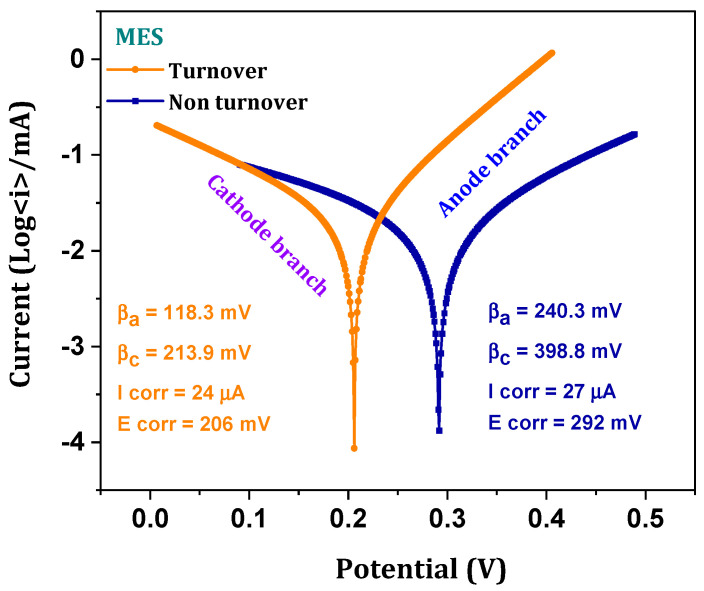
Tafel diagrams of turnover and non-turnover operations.

Electrode kinetics and biocompatibility of the electrodes were evaluated through the Tafel plots (drawn from the LSV) based on the Butler–Volmer equation [22,25,26,27,28,29] (Figure 6). Log integration of the generated reduction current in LSV depicts the kinetics of the charge transfer, which accounts for the substrate (Syngas) utilization and product formation [22]. Oxidative (βa) and reductive (βc) slope values of 240.3 mV/dec and 398.8 mV/dec were observed, respectively, in the non-turnover condition, which decreased (118.3 mV (βa); 213.9 mV (βc)) with the turnover operation indicating higher transfer of charge from the working electrode. In order to avoid errors in the kinetic charge transfers, blank Tafel slopes were drawn from the abiotic LSV. As relations between the slope constants and reduced equivalents transfer are inverse, the higher constant slope values represent the lower charge transfer efficiency. This is well correlated with system C1 gas fermentation performance [22]. Redox slope constant values describe the nature and efficiency of the electrochemical process that occurs in the MES. Bidirectional transfer of the charged species from the electrode to catalyst/electrolyte was increased when the constant slope resistance was decreased. Lowering the redox slope constant values in biotic operation suggests the increased electrocommunication between the biocatalyst and biocathode [25]. Higher charge communication accounts for the overall fermentation performance of the systems by consuming the transferred charge species. Higher product synthesis was observed in the MES, where lower slope constants were recorded. Lower the reduction (βc, 213.9 mV/dec) and oxidation (βa, 118 mV/dec) slope constants in turnover plots showed the tendency of biocathode towards reduction (in syngas) and the oxidation of CO to CO_2_, respectively. Detection of both oxidized and reduced species in the CV relates to these decreased slope constant tendencies. In addition to charge transfer kinetics, the biocompatibility of the electrodes was analyzed by taking the corrosion properties derived from the Tafel corrosion analysis as a marker. Corrosion currents (Icorr) and corrosion potential (Ecorr) attributes to the deteriorated characteristics of the electrodes. Lower corrosion current (Icorr = 24 µA; Ecorr = 206 mV) of biocathode than the non-turnover (Icorr = 27 µA; Ecorr = 292 mV) operation can be able to show the stability and biocompatibility of the biocathode [22,25]. Lower the corrosion rates (currents and potential) may also depict the persistent conversion of the biocathodes, which might also assist the rate of fermentation.

### 3.6. Electrochemically Active Substrate Profile

**Figure 7:**
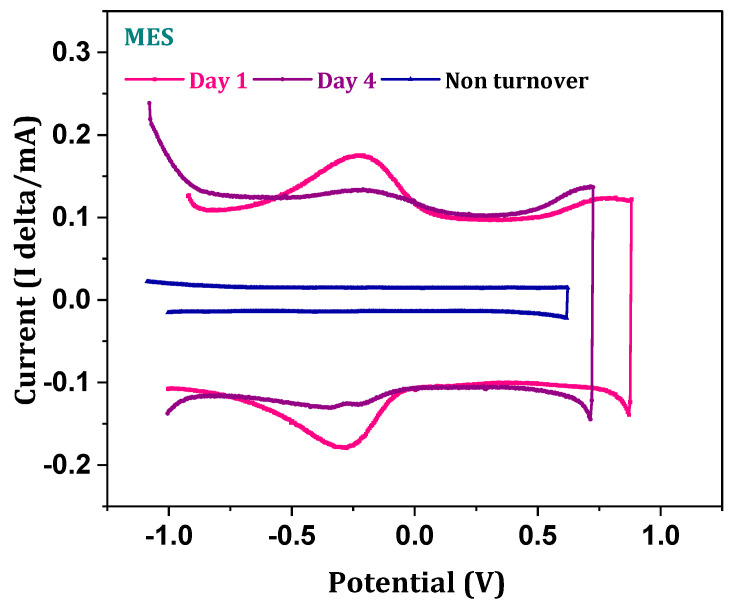
Differential pulse voltammogram plots (initial and 4th day).

Electrochemically active substrate fate during the fermentation process was further investigated by the differential pulse voltammograms (DPV). The pulse wave form of the potential results in the current response generated by the electrochemically active components in the system. The proportional relation between the generated peaks and electrochemical substrate suggested higher peak currents representing the higher concentration of substrate [30,31]. Non-turnover DPV showed a straight line indicating the absence of electrochemically active substrate, whereas the turnover voltammogram depicted the symmetric peaks near the −0.31 V with 1.8 mA current intensity (Figure 7). The distinct peak at 0.3 V represents the presence of electroactive carbonic ion concentration in the electrolytes. Dissipation of the gaseous substrates into the electrolyte results in the formation of carbonic and bicarbonate ions. Peak intensity on the first day was high, which reduced at the end of cycle operation (1.1 mA), indicating the reduction in the concentration of the substrate over the period of operation. A decrease in the electrochemically active substrate in MES proportionally correlates with increased product formation, inorganic carbon fixation and conversion efficiencies by the biocatalyst.

### 3.7. Internal Resistance-Charge Transfer Mechanics

**Figure 8:**
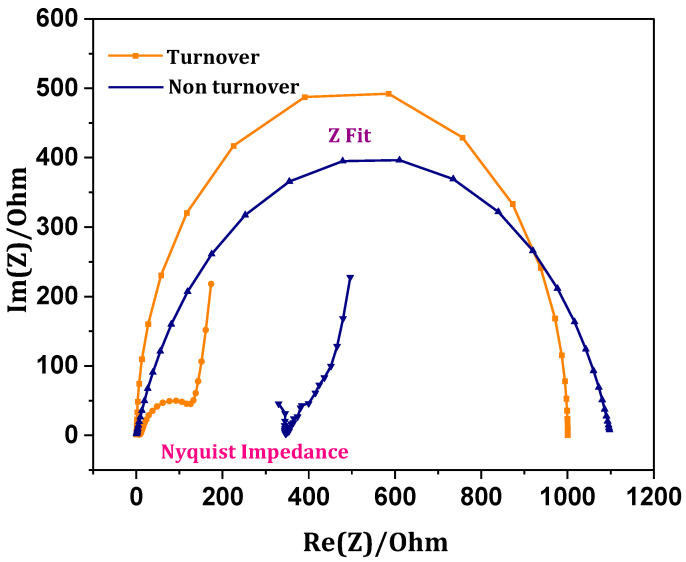
Comparative impedance and Z-fit of MES (non-turnover and turnover).

Charge transfer becomes influenced by electrolytes and electrodes, which include characteristic features such as individual ohmic resistance [23,31]. Electrochemical impedance spectroscopy (EIS) provides the semicircle plots representing the cumulative internal resistance. Blank EIS was performed, and Nyquist impedance was plotted to study the ohmic (Ω_ohm_) and charge transfer (Ω_ct_) resistance (Figure 8). A higher radius of the non-turnover Z fit represents more charge transfer resistance (Ω_ct_ 1.1 kΩ), whereas the Z fit at the end of the experimental operation showed a decrease in the semicircle radium with narrow charged transfer resistances (Ω_ct_ 0.8 kΩ). Higher charge transfer resistance hinders the charge diffusion from the electrode to the electrolyte. Ohmic resistance (Ω_ohm_) of the abiotic operation depicts a higher resistance of 500 Ω than the biotic operation (16.8 Ω). Reduced charge transfer and ohmic resistances of biocathode depict the effective flux of charged species, which assist the growth of the electroactive biofilm on the cathode. Increased growth of biocatalyst on the working electrode surface triggers an efficient reduction in the syngas by higher electron flux with the feasibility of forming products of interest. Higher charge communication minimizes the internal charge transfer resistances that facilitate the active metabolism of the electroactive bacteria [32]. Lower charge losses influence the performance of the system by promoting electroactive microbial growth [23]. Lesser resistances of the biocathode at the end of operation indicated the active adaptation of the cultures towards the redox environment. An increase in the fermentation product (acetate/ethanol) titer at the end of the experimental operation relates to a decrease in resistances/impedances of the MES.

### 3.8. Cathodic Reduction Responses

**Figure 9:**
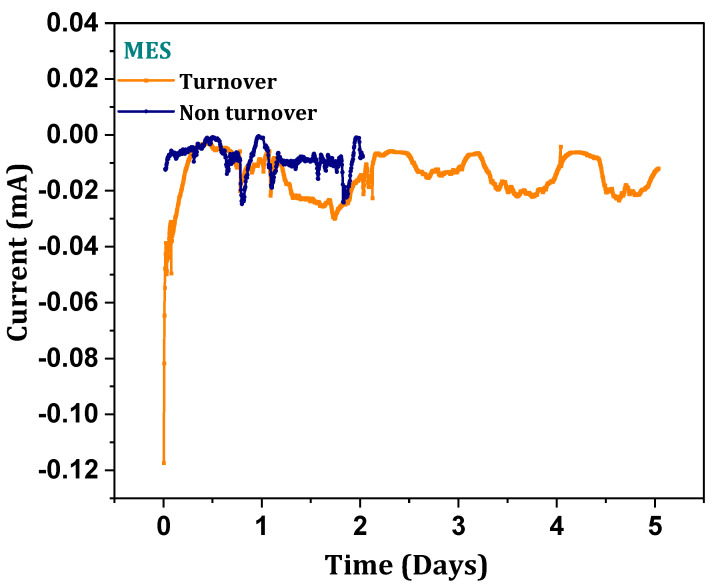
Reductive catalytic current generations (turnover and non-turnover).

Chronoamperometry (CA) was recorded against the applied potential with function time. Applied potential on the working electrode drives the electrochemical reduction in inorganic carbon of syngas in response to the catalytic current. The current profile (marginal) recorded in the abiotic operation might be due to the non-faradaic electrical double layer current (Figure 9). In order to infer the reductive capabilities of abiotic operation, amperometry was performed with an applied potential of −0.5 V. Reductive catalytic current (RCC) generations were recorded with an average of −0.03 mA. Generated reductive currents illustrate the syngas reduction tendencies [14,21,22]. Consistent charge transfer from the cathode surface to the outer membrane electroactive components of the microbe depicts the substrate reduction capability of the carboxydotrophic biocatalyst [14,22]. Continuous syngas sparging into the medium with optimal flow rate enabled the electrogenic activity of the biocatalyst towards product formation by consuming the externally discharged charge. Generated reductive catalytic currents due to the poised potential depicts the proportional electrochemical activities of the biocatalyst, which correlated with substrate conversion and fixation efficiencies. Generated reductive catalytic current utilization towards the fermentation products reflects the capacity of charge utilization by biocatalyst [33,34,35,36]. Coulombic consumption efficiency (CE) and electron recovery in fermentation products were calculated. The ratio to provide coulombs in terms of reductive current on the working electrode that is involved in the product was calculated using the Equation (5)

(5) CE (%)=b∗n∗F/CT

where b is the number of electrons transferred in the products (eight in the case of acetate), n is the number of moles of fermentation product, and F is the faraday constant (96,485 C·mol^−1^). The CT total coulombs are calculated by integrating the current generated. Specific to acetate production, the Coulombic efficiency of 111.6% was observed with syngas feedstock. The higher Coulombic efficiency indicated the effective charge transfer from the reductive catalytic current to fermentation products. Triple charge availability, i.e., in the form of reductive current, oxidation of CO and gaseous H_2_ fraction in the syngas mixture, led to higher Coulombic efficiency than usual (100%) [24,37,38,39].

### 3.9. Microbial Community Distribution

**Figure 10:**
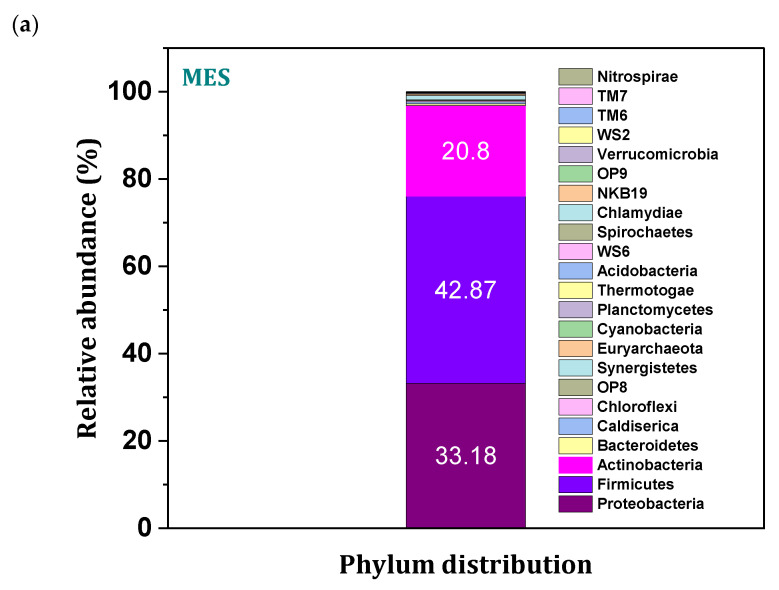

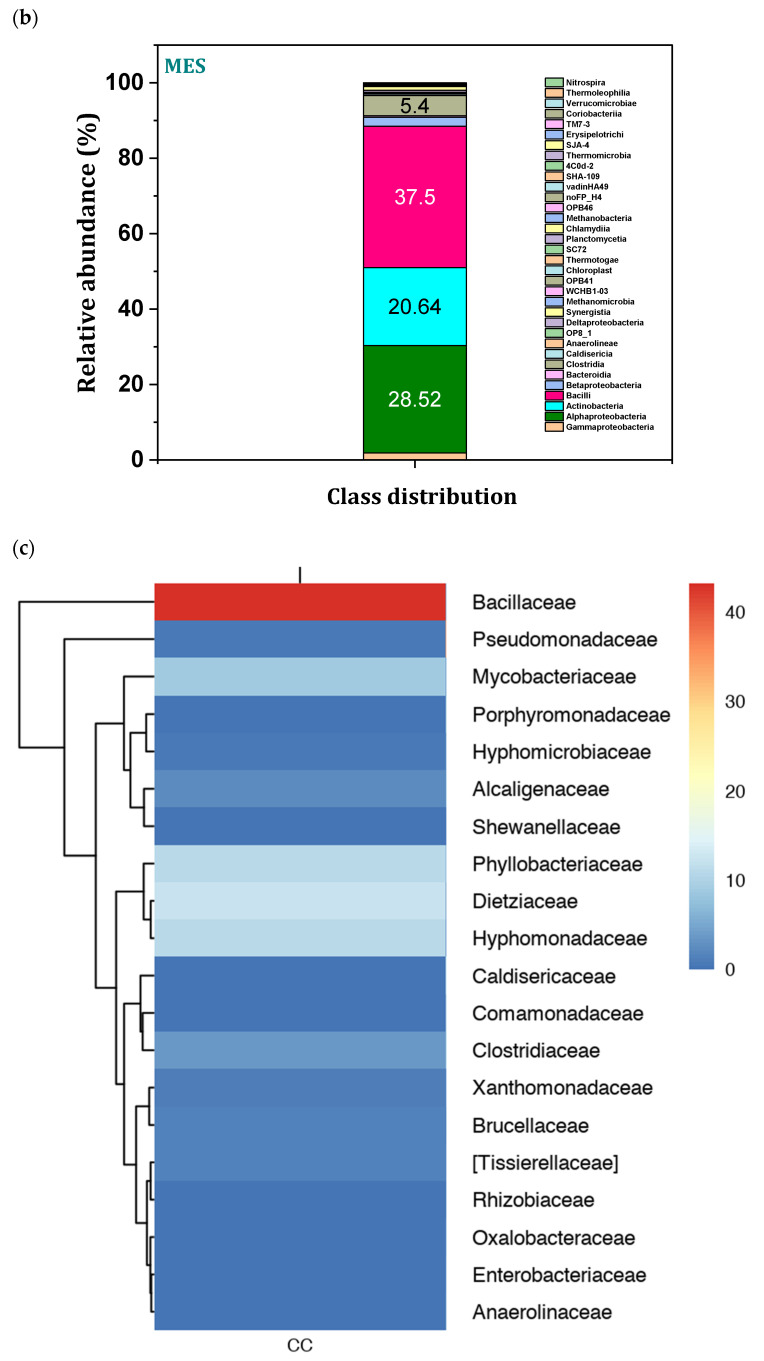

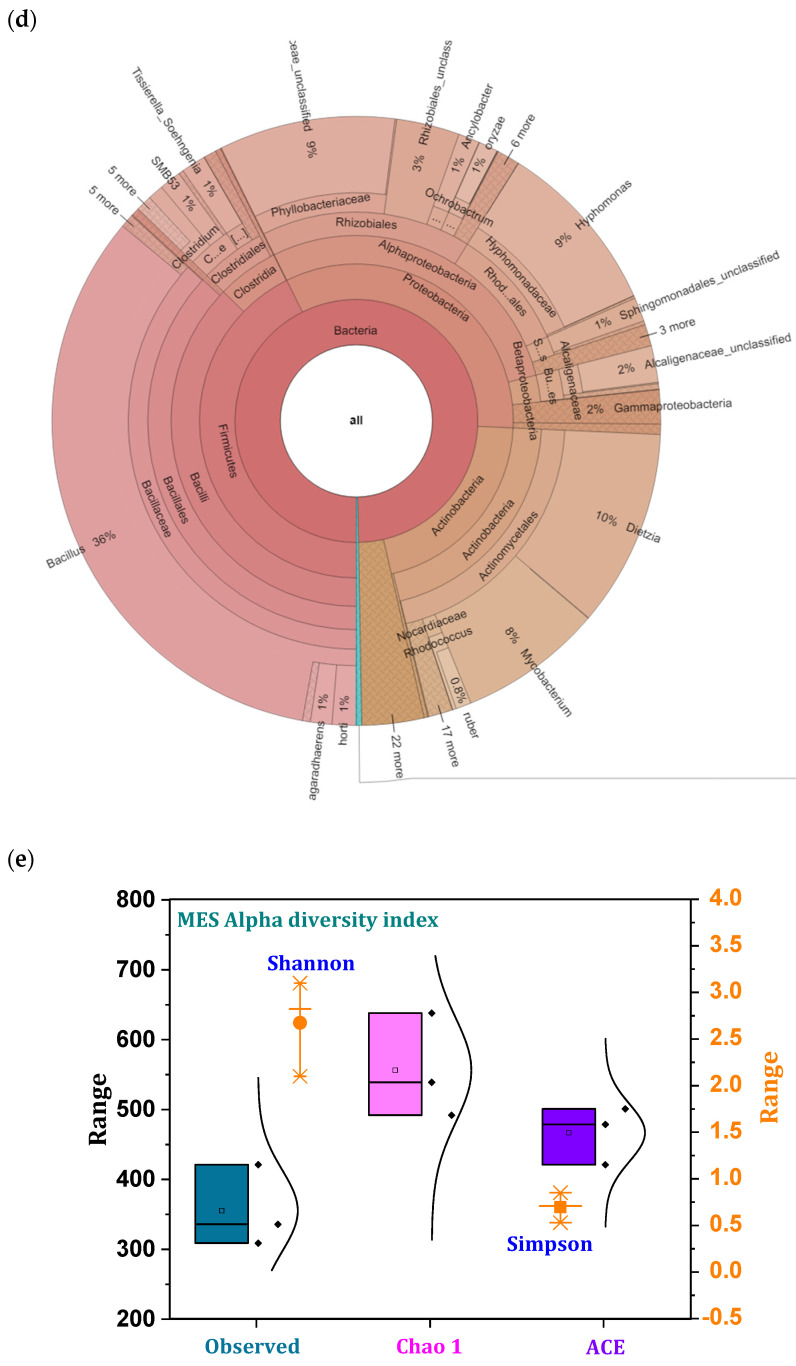
(**a**) Relative abundance of microbial community in phylum level; (**b**) class distribution; (**c**) family level; (**d**) krona plot representing the total community; (**e**) normal boxplots of alpha diversity.

A total number of 99,302 amplicon reads (OTU) were obtained, with an average length of 301 base pairs with 55% GC content. Phylum level distributions of the microbes in the enriched culture revealed the dominance of Firmicutes, Actinobacteria and Proteobacteria with a relative abundance of 42.87, 33.18 and 20.8%, respectively (Figure 10a), followed by Bacteroidetes, Acido bacteria and Chloroflexi distribution. Phylum abundance directly evidenced the efficient syngas fermentation to acetate with the presence of the gene set encoded for different biological steps of acetyl-CoA reduction pathway in the spore-forming phylums such as Firmicutes and Actinobacteria [40,41]. Insights into the gene lineages responsible synthesis of acetyl CoA were encoded in the Actinobacteria phylum that highly contributed to the conversion efficiency of inorganic carbon fractions of syngas into the WLP metabolites [40]. Loci of the hydrogenase genes account for the redox reactions of the molecular hydrogens and release the charged molecules for the reduction in C1 gases owned by the actinobacteria cultures [41]. Enriched cultures of MES reveal the presence of the Actinobacteria phylum with a fraction of 33.18%. Evidence of the higher substrate conversion rates with increased Coulombic efficiencies was related to the presence Actinobacteria phylum, which accounted for the higher fermentation rates by providing adequate reducing equivalents through enzymatic functions [40,41]. Further study on the diversified taxonomic profile of cultures provided a phylum level synergy between the identical communities (oxidation by Proteobacteria and reduction by Firmicutes) in converting the supplemented gaseous feedstock. Mediated and Direct interspecies electron transfer (DIET/MIET) might have attributed to the synergy of microbial communities accounts for the high substrate conversion rates. The appearance of several redox species in the electrochemical study might assist the charge communications through DIET/MIET between the diversified microbiota Acid bacteria phylum plays a critical role in further synthesis of acetate from acetyl-CoA, which was synthesized by the homoacetogenic phylum (Firmicutes) [41].

Class-level taxonomic distributions represent the relative dominance of Bacilli (37.5), Alpha proteobacteria (28.52), Actinobacteria (20.63), Clostridia (5.4) (Figure 10b) and others in minor fraction. Class Bacilli belonging to the Bacillota (Firmicutes) phylum has the capability to fix the inorganic carbon in non-photosynthetic routes [42]. Gene loci of the carbon monoxide dehydrogenase (CODH) and acetyl Co-A synthase (ACS) are predominant in the microbial families of Bacillaceae and Clostridiaceae (Figure 10c,d), which account for the C1 fixation [40]. Involvement of identical clostridial class microbes (Krona analysis chart) evidenced the accumulation of acetate and ethanol as a result of clostridial metabolism. The presence of C1 fixing microbial diversity in different taxonomical order assists the biosynthesis of the acetate and ethanol through WLP. The distribution of carboxydotrophic community in the system evidenced the synthesis of fermentation products and syngas fixation/conversions.

The intercommunity difference was further investigated by the α-diversity of the biocatalyst (Figure 10e). Three kinds of α-diversity indices (Observed, Shannon and Simson) were analyzed to the culture and compared to predict variance spread in the community. Observed α-diversity ranges between 300 and 420 in the duplicate analysis of the samples. Lower Simpson (2.5) and Shannon (0.6) index values were observed in the enriched culture, evidenced by the distributions of the selected community dominance that drives the specific metabolic reactions. Low variance and evenness distributions of the carboxydotrophic microbes account for the specific acetogenic and solventogenic process in which syngas feedstock converted into the WLP pathway metabolites account for the microbiome assisting the dominant growth of the specific microbiome (C1-fixing) [43]. Cho1 and abundance-based coverage estimators (ACE) species richness was depicted in the median of 530 and 480, respectively. Low indices of the α-diversity, Cho1 and ACE represent the selective abundance in the diversity of the mixed culture [40].

## 4. Conclusions

A single-chambered MES system with carbon cloth (CC) as the working electrode was studied for syngas fermentation employing enriched carboxydotrophic consortia with a semi-continuous operation (batch mode operation with continuous gaseous sparging in the electrolyte). CC provides an adequate surface for biofilm formation within a short period. Higher acetic and ethanol product titers in the medium after the third cycles of operation correlate well with the biofilm formation. Electrochemical parameters of the MES revealed the reductive capabilities of the biocatalyst, and microbe with low charge transfer resistance with higher Coulombic efficiencies enabled the cathodes biocatalyst communications towards syngas conversion. An abundance of syngas fixing microbe phylum and class abundance (Actinobacteria, Firmicutes/Clostridia and Bacilli) assist the syngas fermentation through gene loci responsible for reduction metabolism. Supplementation of syngas in regulated flow into the electrolyte of designed MES enables bioconversion of the CO_2_/CO.

## Data Availability

Not applicable.
